# Evaluating the role of coherent delocalized phonon-like modes in DNA cyclization

**DOI:** 10.1038/s41598-017-09537-y

**Published:** 2017-08-29

**Authors:** Ludmil B. Alexandrov, Kim Ø. Rasmussen, Alan R. Bishop, Boian S. Alexandrov

**Affiliations:** 1 Theoretical Division, Los Alamos National Laboratory, Los Alamos, 87545 United States of America; 2 University of New Mexico Comprehensive Cancer Center, Albuquerque, NM 87102 USA

## Abstract

The innate flexibility of a DNA sequence is quantified by the Jacobson-Stockmayer’s J-factor, which measures the propensity for DNA loop formation. Recent studies of ultra-short DNA sequences revealed a discrepancy of up to six orders of magnitude between experimentally measured and theoretically predicted J-factors. These large differences suggest that, in addition to the elastic moduli of the double helix, other factors contribute to loop formation. Here, we develop a new theoretical model that explores how coherent delocalized phonon-like modes in DNA provide single-stranded ”flexible hinges” to assist in loop formation. We combine the Czapla-Swigon-Olson structural model of DNA with our extended Peyrard-Bishop-Dauxois model and, without changing any of the parameters of the two models, apply this new computational framework to 86 experimentally characterized DNA sequences. Our results demonstrate that the new computational framework can predict J-factors within an order of magnitude of experimental measurements for most ultra-short DNA sequences, while continuing to accurately describe the J-factors of longer sequences. Further, we demonstrate that our computational framework can be used to describe the cyclization of DNA sequences that contain a base pair mismatch. Overall, our results support the conclusion that coherent delocalized phonon-like modes play an important role in DNA cyclization.

## Introduction

The flexibility of the DNA molecule plays an important role in a multitude of biological functions as well as in the compact storage of the genetic material of cells^1^. For example, sharply bending DNA around nucleosomes is used to tightly pack the genome in each eukaryotic cell^2^, while DNA bending by transcription factors is a common cellular mechanism that participates in regulation of gene expression^3–6^. There have been numerous experimental and theoretical studies examining the thermodynamics of DNA bending^7–26^. In the majority of cases, bending of DNA results in loop formations with lengths shorter that DNA persistence length^27^. Such loops contradict the classical polymer physics view, in which double-stranded DNA (dsDNA) is virtually unbendable at scales below its persistence length.

Due to its long persistence length of approximately 150 base pairs (bp), dsDNA has typically been modeled as an elastic rod with mechanical properties well described by the wormlike chain model (WLC)^28^. Within the WLC model, the conformational properties of a sequence depend entirely on the notion of a persistence length, and hence, DNA loops with lengths shorter than 150 bp are energetically extremely costly and the probability for their spontaneous thermodynamic creation is vanishingly small^29^. As such, the WLC model’s prediction of Jacobson-Stockmayer’s J-factors (which measures the propensity for DNA loop formation, see ref. 30) for short DNA sequences is in a sharp contrast to several *in vivo* and *in vitro* experimental observations^9–16^. Thus, the basic WLC model is unable to accurately characterize the cyclization of short DNA fragments, although it consistently describes the cyclization of long DNA segments.

The basic WLC model ignores both the nucleotide sequence and the three-dimensional (3D) helical structure of DNA, both of which are known to affect cyclization rates. For example, ignoring the 3D nature of DNA results in disregarding the proper torsional orientation of DNA segments, and hence does not allow describing the experimentally observed oscillations of the cyclization rates due to the approximately 10 bp torsional period of the double helix^31^. Further, the lack of nucleotide sequence and intrinsic curvature information in the basic WLC model limits its predictive capability, since experimental studies have shown that periodic stretches of consecutive adenine-thymine base pairs possess curved equilibrium conformations^32^. Such periodic DNA sequences exhibit a natural sequence-dependent static bending, also known as intrinsic curvature, that can result in higher cyclization rates^33^.

Incorporating the 3D structure and sequence-dependent intrinsic curvature of DNA allows a better representation of the nature of the elasticity of the double helix^34^. As a first approximation, the intrinsic curvature can be considered as an equilibrium property of each DNA segment^35^. To more realistically calculate cyclization properties of DNA, a coarse-grained Monte Carlo approach incorporating the 3D structure, intrinsic curvature, and DNA sequence was developed by Levenet, Crothers, and Zhang^36, 37^, by Manning, Maddocks, and Kahn^38^, and by Czapla, Swigon, and Olson^39^.

Recently, we applied the Czapla-Swigon-Olson (CSO) model^39^ to perform an extensive characterization of the effect that distinct sets of structural parameters have on estimating J-factors of DNA sequences with different lengths^24^. Our analysis encompassed seven sets of structural and conformational parameters to calculate the J-factors of 86 DNA segments with experimentally measured J-factors, with lengths between 50 bp and 330 bp, as well as of 20,000 *in silico* generated random sequences, with lengths between 350 bp and 4,000 bp. Our analysis revealed that a set of parameters based on an examination of nucleosome positioning^40^ provides results most consistent with the experimental measurements. Importantly, we showed that all sets of structural parameters perform well for the *in silico* generated sequences longer than ~350 bp and that the choice of structural parameters and the nucleotide sequence of the DNA segment makes little difference in estimating the cyclization of these long sequences. In contrast, all investigated sets of structural parameters performed poorly when used for estimating J-factors of DNA segments shorter than ~100 bp, yielding predictions with up to six orders of magnitude difference compared to experimental findings. The inability to describe the cyclization rates of ultra-short DNA sequences suggests that taking into account the intrinsic curvature and 3D structure of DNA fails to capture the salient physics for short DNA segments.

In this paper, we develop a new computational approach for evaluating DNA cyclization by linking the CSO model^39^ with our Extended Peyrard-Bishop-Dauxois (EPBD) approach^41–43^. The CSO model accounts for DNA intrinsic curvature and 3D structure, while the EPBD approach accurately describes DNA opening dynamics that produces local single-stranded regions (i.e., DNA bubbles) in the double helix^44^. This coupled CSO-EPBD model is applied to 86 DNA sequences with experimentally characterized J-factors previously examined in ref. 24 (Supplementary Table S1). Our analysis demonstrates that, without changing any of the original parameters in the CSO model or the EPBD model, the coupled CSO-EPBD model is able to accurately determine the J-factors of ultra-short DNA sequences with most predictions being within an order of magnitude of experimental measurements. Further, the coupled CSO-EPBD model continues to accurately describe the J-factors of longer sequences, and we also demonstrate that this model is applicable to DNA sequences containing a base pair mismatch.

## Results

### Coherent delocalized phonon-like modes and DNA looping

The structural integrity of biological macromolecules is primarily governed by hydrogen bonds (H-bonds), which have natural vibration frequencies in the terahertz range^45, 46^. H-bonds are much weaker (~few *k*_*B*_ *T* s) than covalent bonds, causing these macromolecules to experience slow conformational motion resulting from the inherent thermal fluctuations at biological temperatures. The DNA molecule’s slow motions induce spontaneous openings and re-closings of the double helix known as “DNA breathing”^47, 48^, or spontaneous “base flipping”^49^, or “DNA bubbles”^45^, or “coherent delocalized phonon-like modes”^45^ (The term “delocalized” refers to the fact that the low-frequency (THz) vibrational modes of the bio-macromolecules are indeed delocalized in the wavenumber space, while they are localized in the real space, forming DNA bubbles that coherently span only a few base pairs). The frequency of occurence of these transient openings depend entropically both on the local sequence and its structure. The propensity for DNA bubbles is related to the local stability and flexibility of the molecule^50^ and it plays a key role in many biological processes such as: reading of the genetic code (transcription)^51–53^, DNA production (replication)^54, 55^, protein-DNA binding^56–60^, DNA methylation^61^, and DNA repair mechanisms^62^.

It has been proposed that the propensity for DNA bubbles and the flexibility of a DNA segment are interrelated^8, 63^. As such, in addition to the elastic moduli of the double helix, loop formations are facilitated by “flexible hinges”. In turn, these “flexible hinges” are due to the fluctuation of DNA base-pair openings resulting in more flexible single-strand DNA (ssDNA) segments in the, otherwise, rigid dsDNA. Thus, the experimental results demonstrating unusually high J-factors for short DNA sequences may be due to spontaneous internal strand-separation. Although energetically expensive and therefore rare, such strand-separation can be entropically favorable and may assist DNA local bending when forming loops between 70 and 100 bp. As explained in ref. 8 (consistent with the values obtained in experiments) the formation of 3 bp ssDNA regions (i.e., DNA bubbles) are associated with a free energy cost of ~6 *k*_*B*_ *T* to 15 *k*_*B*_ *T*. Hence, modified WLC approaches incorporating DNA’s ability to spontaneously develop local ssDNA regions, realized via parameters for a phenomenological density of hinges or kinks, have been explored with some success^8, 13, 19^. However, these models largely ignored the DNA sequence as well as the intrinsic curvature and 3D structure of the double helix.

Developing a predictive understanding mandates a more specific ability to characterize the sequence-dependent dynamics of the transient opening and re-closing of dsDNA while also accounting for DNA’s intrinsic curvature and 3D structure. Here, by combining the CSO and EPBD models, we develop such an approach and evaluate its predictive capabilities on DNA sequences with previously measured J-factors.

### Description of the CSO-EPBD model

The CSO model (see Methods) utilizes equal standard variations for the fluctuations of the tilt and roll angles (i.e., isotropic bending). The model uses a 147 bp persistence length for dsDNA and, as such, mandates a root-mean-square fluctuation of 4.84° for the tilt and roll angles. In contrast, the CSO model standardly uses 4.09° as the root-mean-square fluctuations for the twist angle. Our CSO-EPBD model builds upon the original CSO model but it also includes coherent delocalized phonon-like modes in DNA, which correspond to entropically-driven single stranded regions in the DNA sequence. These single stranded regions are more flexible than dsDNA and act as “flexible hinges” in the otherwise rigid dsDNA molecule since the persistence length of single stranded DNA (ssDNA) is only ~3 bp^64^. To incorporate the ssDNA persistent length in our CSO-EPBD framework, the root-mean-square for the roll and tilt angles were set to ~30.08°, while ~27.96° was used for the root-mean-square of the twist angle (both consistent with the assumptions in ref. 39). Note that, in this study, we do not use the naturally straight DNA model that was one of the models proposed in the original CSO publication^39^. Rather, we use the CSO model with experimentally measured parameters to account for presence of static curvature in the DNA fragments.

Our EPBD model (see Methods) describes the transient base-pair openings of the double helix by quantifying the probability for each base pair to be in a single-stranded state and hence to act as a hinge. As such, for each examined DNA sequence, we generated a profile that captures the probability for each base pair to be in a single-stranded state due to thermal fluctuations of the double helix.

The EPBD probability profiles (Supplementary Table S2) were imbedded in the CSO model by using a Markov Chain Monte Carlo approach. For each base pair in a sequence, a number was generated from a uniform random distribution between 0 and 1 and this number was compared to the EPBD opening probability for this base pair. When this random number was lower than or equal to the EPBD opening probability for this base pair, the DNA base-pair was considered to be in a single-stranded state and the root-mean-square values for ssDNA were used for this base pair when generating a particular chain. Conversely, when the random number was higher than the EPBD opening probability, the DNA base-pair was considered to be in a double-stranded state and the root-mean-square values for dsDNA were used for this base pair. In all cases, we used the same set of structural DNA parameters previously shown to yield optimal results when compared to experimental measurements (Supplementary Table S3). It is important to note that by associating the EPBD-generated opening profiles with the hinge density in the formulation of Yan and Marco^8, 65^, we obtained (by using Eq. (33) in ref. 65) that the free energy of the transient double-strand openings, calculated by EPBD derived probabilities, requires on average ~10 *k*_*B*_ *T*, in agreement with arguments made in refs 10 and 65.

### Applying the CSO-EPBD model to DNA sequences with different lengths

**Figure 1:**
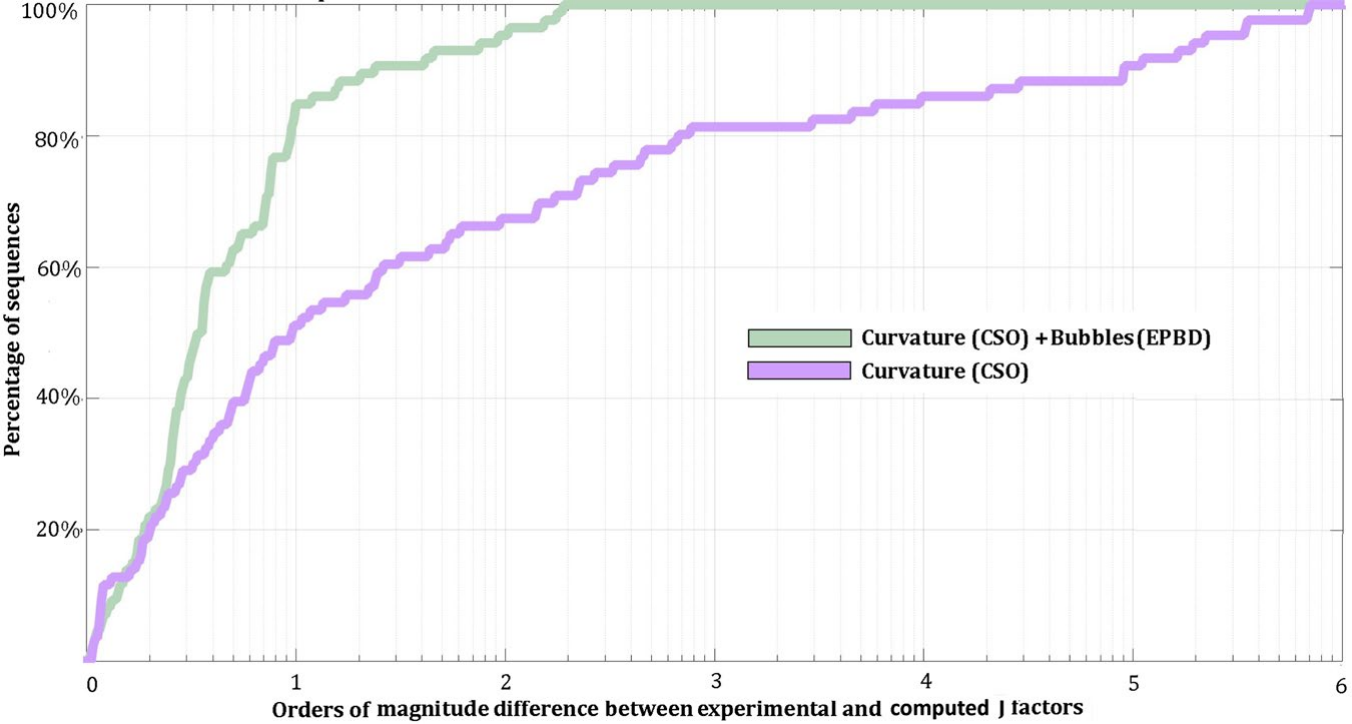
Estimated J-factors for all examined DNA sequences. The two different curves with colors correspond to the CSO and CSO-EPBD model calculations. The y-axis reflects the orders of magnitude difference between experimentally measured and computationally derived J-factors. The x-axis corresponds to the percentage of sequences for a given order of magnitude difference.

Our implementation of the new CSO-EPBD model was used to calculate the J-factors of 86 DNA sequences with experimentally determined J-factors and lengths between 50 bp and 325 bp. Comparisons were performed between experimentally determined J-factors, J-factors derived using the original CSO model, and J-factors derived using the CSO-EPBD model (Supplementary Table S4). These comparisons show that the J-factors calculated using the CSO-EPBD model are much closer to experimental measurements compared to the J-factors calculated using the original CSO model (Supplementary Table S4). To quantify the differences between the two *in silico* calculated J-factors and the experimental measurements, we calculated the percentage of sequences for which their computationally estimated J-factors are within a particular absolute distance from the actual experimental measurements (Fig. 1). The results based on the CSO-EPBD model are much closer to experimental measurements: ~85% of J-factors are within an order of magnitude of experimentally measured values. In contrast, only ~51% of the J-factors calculated by the original CSO model are within the same range.

**Figure 2:**
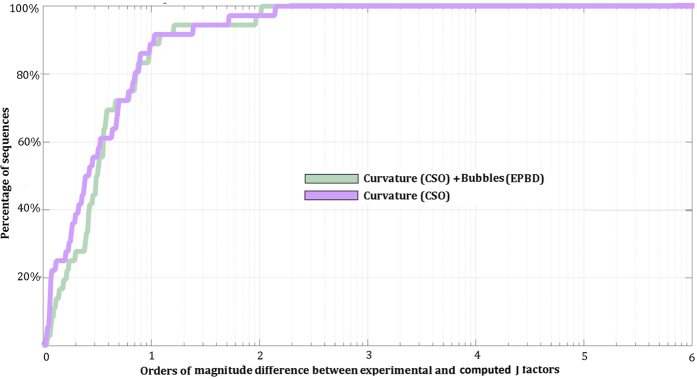
Estimated J-factors for all sequences with lengths longer than 100 bp. The two different curves with colors correspond to the CSO and CSO-EPBD model calculations. The y-axis reflects the orders of magnitude difference between experimentally measured and computationally derived J-factors. The x-axis corresponds to the percentage of sequences for a given order of magnitude difference.

**Figure 3:**
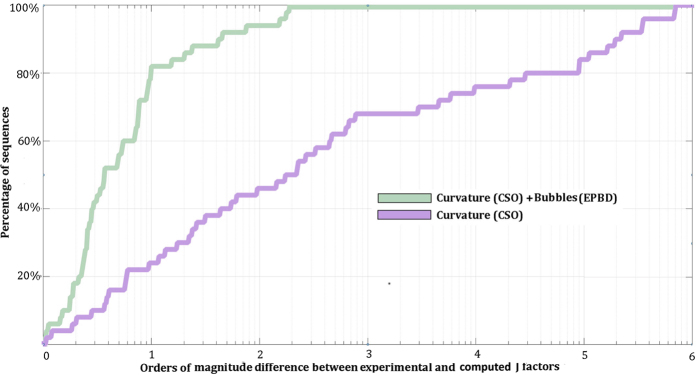
Estimated J-factors for all sequences with lengths shorter than 100 bp. The two different curves with colors correspond to the CSO and CSO-EPBD model calculations. The y-axis reflects the orders of magnitude difference between experimentally measured and computationally derived J-factors. The x-axis corresponds to the percentage of sequences for a given order of magnitude difference.

Importantly, both the CSO-EPBD model and the original CSO model perform equally well for sequences with lengths longer than 100 bp (Fig. 2). However, the original CSO model is unable to accurately describe J-factors of sequences shorter than 100 bp since less than ~25% of sequences are within an order of magnitude of experimental measurements (Fig. 3). In contrast, the results from the CSO-EPBD model are within an order of magnitude for ~82% of all examined ultra-short sequences. Further, the J-factors of the remaining ~18% of ultra-short sequences are within approximately two orders of magnitude from the experimental measurements. In contrast, J-factors calculated by the CSO model for many sequences are four, five, and even six orders of magnitude smaller than the experimental results. Overall, these results support that our CSO-EPBD model provides a significantly better description of DNA cyclization for ultra-short segments compared to the original CSO model (Fig. 3). It is also noteworthy that the CSO-EPBD model is able to describe the J-factors of ultra-short DNA sequences almost as well as the ones of long DNA sequences: ~82% of ultra-short versus ~89% of long sequences are within an order of magnitude of experimental measurements.

### Applying the CSO-EPBD model to a DNA sequence with a base pair mismatch

One limitation of most previous models is that they cannot be used to examine the cyclization of a DNA sequence containing a specific nucleotide modification, for example, a mismatched base pair. In contrast, our new CSO-EPBD model can be used to calculate the J-factors of DNA sequences with the presence of nucleotide modifications. Here, our EPBD simulations take into account a sequence mismatch by assigning a probability of one to observe this base pair open in the profile of a DNA sequence (i.e., the mismatch is always in a single-stranded state; see Supplementary Table S5). To evaluate the ability of CSO-EPBD to estimate the J-factors of sequences containing a mismatch base pair, we applied our model to a DNA fragment that was previously experimentally characterized for both having a C:C mismatch in the middle of the sequence as well as not having such a mismatch^15^. The J-factor of the sequence with a C:C mismatch, calculated by CSO-EPBD, is 4.99E-10, which is more than an one order of magnitude increase compared to the J-factor of the sequence without a mismatch (J = 3.32E-11). Comparison between the calculated J-factors by CSO-EPBD and previously performed experimental measurements^15^ reveals good agreement for both cases.

## Discussion

In this study, we developed a new theoretical model for describing DNA cyclization by combining the EPBD model with the CSO model. This CSO-EPBD model was used to perform an extensive examination of the effect that sequence dependent coherent delocalized phonon-like modes of DNA have on estimating J-factors of DNA sequences with different lengths. We applied our implementation of the new CSO-EPBD model to 86 DNA segments with experimentally characterized J-factors, with lengths between 50 bp and 325 bp. Our analysis demonstrates that the CSO-EPBD model yields results in good agreement to experimental measurements. Further, even for sequences with lengths less than 100 bp, the CSO-EPBD model performs very well, yielding more than 82% of the calculated J-factors within an order of magnitude of experimental measurements. The analysis of J-factors for sequences longer than 100 bp shows that the CSO-EPBD model gives results indistinguishable from the results obtained by the CSO model alone.

Even though the calculated J-factors are significantly improved and within an order of magnitude from experimentally measured values, the results derived by leveraging the CSO-EPBD model still differ from the exact experimental measurements for ultra-short sequences. This suggests the need to explore more accurate models for DNA breathing and DNA curvature in the future. One possible explanation for these differences is that the set of structural parameters used in CSO-EPBD is not sufficiently accurate for describing some of the sequences. Another likely explanation is that the EPBD calculated average opening profiles neglect the effect of adjacent base pairs opening in a correlated fashion. Future studies taking into account the specific, rather than the mean, structural parameters of a DNA sequence and incorporating DNA breathing dynamics directly into the CSO model may be needed.

There are other factors that could be affecting our results. Our examination relies on previous measurements of J-factors and structural parameters. However, these experimentally derived values exhibit standard errors that were ignored in our analysis and the mean values were assumed to be representative. Further, parts of the experimental data analyzed in this paper have been considered problematic by some researchers^66^. Nevertheless, assuming the accuracy of the experimentally measured J-factors as well as the mean values of the used structural parameters, the present study provides a systematic evaluation of the role of DNA bubbles in calculating cyclization rates.

Importantly, the present work, for the first time, supports a direct link between sequence-dependent bubbles and more accurate calculation of J-factors for short DNA segments. In addition, considering recent experimental observations^45^ demonstrating that DNA bubbles are indeed in the terahertz range leads to an exciting avenue for future exploration of J-factors in the presence of a strong, pulsed terahertz field. Such future studies will allow elucidation of the connection between terahertz irradiation and DNA functionality^67^.

## Methods

### The EPBD model

To describe the bubbles in DNA, here, we use the EPBD model of DNA^43^, which is an extension of the original Peyrard-Bishop-Dauxois (PBD) DNA model^41, 42^ that includes sequence-specific stacking potentials. The EPBD model is a quasi-two-dimensional nonlinear model that describes the transverse opening motion of the complementary strands of double-stranded DNA, while distinguishing the two sides (left - *v*_*n*_ and right - *u*_*n*_) of the DNA double strand, and can describe the presence of nucleotide modifications. The potential of the EPBD model, *V*_*EPBD*_, is (see, e.g., ref. 59)

1 $${V}_{EPBD}=\sum _{n=1}^{N}\,U({u}_{n};{v}_{n})+W({u}_{n-1},{u}_{n};{v}_{n-1},{v}_{n}),$$

where,

2 $$U({u}_{n};{v}_{n})={D}_{n}{({e}^{-{a}_{n}({u}_{n}-{v}_{n})}-1)}^{2},$$

and,

3 $$\begin{array}{rcl}W({u}_{n-1},{u}_{n};{v}_{n-1},{v}_{n}) & = & \frac{{K}_{n,n-1}^{u}}{2}{({u}_{n}-{u}_{n-1})}^{2}+\frac{{K}_{n,n-1}^{v}}{2}{({v}_{n}-{v}_{n-1})}^{2}\\  &  & +\,\frac{\rho }{4}{e}^{-\beta [({u}_{n}-{v}_{n})+({u}_{n-1}-{v}_{n-1})}\,(\sqrt{{K}_{n,n-1}^{u}}({u}_{n}-{u}_{n-1})\\  &  & {-\sqrt{{K}_{n,n-1}^{v}}({v}_{n}-{v}_{n-1}))}^{2}.\end{array}$$

Here, the sum is over all *N* base pairs of the DNA sequence. For each base pair, *V*_*EPBD*_ includes two degree of freedom; *u*_*n*_ and *v*_*n*_ representing the relative displacement from the equilibrium of the corresponding nucleotide, located, respectively, in the right or left strand of the double helix. These displacements quantify the transverse stretching of the hydrogen bonds between complementary nucleotides. The first term, *U*(*u*_*n*_; *v*_*n*_), is a Morse potential for the n^*th*^ base pair that represents the combined effects of the hydrogen bonds between the complementary bases and electrostatic repulsion of the backbone phosphates. The parameters *D*_*n*_ and *a*_*n*_ depend on the nature of the base pair (A-T versus G-C, i.e., two hydrogen bonds versus three hydrogen bonds). The second term represents a quasi-harmonic approximation of the stacking interactions between consecutive nucleotides, which influences their transverse stretching motion. The exponential term effectively decreases the stacking interaction when one of the nucleotides is displaced away from its equilibrium position, e.g., when one of the nucleotides is out of the DNA stack. The stacking force constants, ${K}_{n,n-1}^{u}$ and ${K}_{n,n-1}^{v}$, depend on: (i) the nature of the nucleotide, (ii) on its closest neighbor, and (iii) on the location of the nucleotide - the right or left DNA strand. The dinucleotide stacking force constants were determined in ref. 43 by comparison with UV-melting curves of DNA oligomers. Here, we exploited EPBD simulations to calculated the opening probability profiles for DNA sequences investigated in ref. 24 using EPBD Markov Chain Monte Carlo (MCMC) simulations (for EPBD parameters and MCMC details see ref. 59). The opening probability derived from MCMC simulations is defined as the fraction of the simulation time a base pair remains open/stretched by more than 2.5 Å, which correspond to a local melting in the EPBD framework. Note that, as we have previously demonstrated^44, 57, 58, 60^, the DNA local breathing/openings of a base-pair depend not only on the closest neighbors but also on the long-distance flanks of that base-pair.

### The CSO model

In the CSO model^39^, each configuration of a DNA segment with *N* base pairs depends on its sequence via the equilibrium (minimum energy) values of the DNA’s standard conformational and structural parameters^68^; twist angle, roll angle, tilt angle, shift displacement, slide displacement, and rise displacement: Θ^0*n*^ = (${\theta }_{1}^{n0}$, ${\theta }_{2}^{n0}$, ${\theta }_{3}^{n0}$, ${\theta }_{4}^{n0}$, ${\theta }_{5}^{n0}$, ${\theta }_{6}^{n0}$). For each, *n* ^*th*^ bp these parameters are defined in relation to the previous (*n* − 1)^*th*^ bp and characterize the static bending. We previously demonstrated^24^ that the CSO model with trinucleotide equilibrium values, Θ^0*n*^, taken from ref. 69 exhibits a superior performance. This study takes the same approach as in ref. 24 and it uses trinucleotide dependencies where, for each *XYZ* trinucleotide sequence, specific values are assigned to the twist angle, roll angle, tilt angle, shift displacement, slide displacement, and rise displacement (Supplementary Table S3) based on the experimental measurements in ref. 40.

The deviations from these equilibrium values, ΔΘ, are caused by the thermal fluctuations and are controlled by the DNA elastic moduli, ${f}_{ij}^{n}$, where, ${f}_{ij}^{n}$ are the elements of the symmetric 6 × 6 elastic force matrix *F*. The energy of each base pair, *G*, in harmonic approximation depends only on these deviations, ΔΘ, and is given by,

4 $${G}_{n}({{\rm{\Theta }}}^{st},F,{{\rm{\Theta }}}^{0n})=\frac{1}{2}\,\sum _{i=1}^{6}\,\sum _{j=1}^{6}\,{f}_{ij}^{n}{\rm{\Delta }}{\theta }_{i}^{n}{\rm{\Delta }}{\theta }_{j}^{n},$$

5 $${\rm{\Delta }}{\theta }_{i}^{n}\equiv {\theta }_{i}^{st}-{\theta }_{i}^{n0}.$$

The equilibrium parameters *θ* ^*n*0^ define the static bending and hence the intrinsic curvature of the DNA segment, while *θ* ^*st*^ are stochastic variables representing the fluctuations. After a diagonalization of the force-constant matrix *F*, the energy for each consecutive base pairs can be written in terms of a diagonal matrix *D* and normal variables *ω*, as: ${G}_{n}=\tfrac{1}{2}{{\rm{\Omega }}}^{T}D{\rm{\Omega }}$. Therefore, the probability for a given configuration becomes,

6 $${P}_{n}({\rm{\Theta }})=\prod _{i}^{6}\,\frac{1}{\sqrt{2\pi \beta {D}_{ii}}}\,{\exp }^{-\frac{\beta }{2}{D}_{ii}{\omega }_{n}^{2}}.$$

This expression represents the probability as a product of independent terms and normal variables that describe the changes of the parameters on the directions of the principal axes of deformation. Hence, to account for the thermal fluctuations, the conformational parameters can be considered as given by normal distributions with specific expectations values and standard deviations: The expectation values define the static bending and are represented by Θ^0*n*^, while the standard deviations define the flexibility and depend on DNA’s elastic moduli. Originally the CSO model assumes that the fluctuations of the tilt and roll angles are equal (isotropic bending) and for the root-mean-square fluctuations of the dsDNA the value of 4.84° is used, corresponding to a persistence length of ~147 bp. Furthermore, the CSO model uses 4.09° as the root-mean-square fluctuations in the twist angle value, which corresponds to a global twisting constant compatible with previously measured equilibrium topoisomer distributions of DNA mini-circles^70^.

Within the CSO model, propensity for cyclization of a specific DNA sequence is estimated by Monte Carlo simulations (for details of the algorithm see refs 24 and 39). The simulations sample the configuration space of the chains obtained by generating series of DNA sequences with structural parameters drawn from normal distributions with given expectation values and standard deviations corresponding to dsDNA. The J-factor that characterizes the cyclization can be determined^36, 37, 39^ as a product of probabilities describing the contribution of the spatial configuration:

7 $$J=\frac{4\pi }{{N}_{A}}W(r\approx \mathrm{0)}\,{{\rm{\Gamma }}}_{r}(\cos \,\gamma \approx \mathrm{1)}\,{{\rm{\Phi }}}_{r,\cos \gamma }(\varphi \approx 0).$$

Here, *W*(*r* ≈ 0) is the probability for a DNA segment to be circular, i.e., to possess the end-to-end distance *r* ≈ 0 (in practice, a threshold of 30*Å* was used). The factor Γ_*r*_(cos *γ* ≈ 1) is the conditional probability that the normal vector of the first and last base pairs are (almost) aligned when the ends of the fragments coincide, i.e., the cosine of the net bending angle is ≈1. In practice, a threshold of cos *γ* > 0.86 was used when *r* ≈ 0. The term Φ_*r*,cos*γ*_(*ϕ* ≈ 0) is the conditional probability that the first and last base pairs coincide. In practice, a threshold of cos *ϕ* > 0.86 was applied when both *r* ≈ 0 and the first and the last base pairs are coplanar, i.e., cos *γ* ≈ 1. The factor $\tfrac{4\pi }{{N}_{A}}$, where *N*_*A*_ is Avogadro’s number, is the normalization associated with the uniformly distributed probability density of bimolecular association. As in our previous works^24^, periodic boundary conditions were used for simulating each sequence. More specifically, the first two base-pairs of each sequence were added to the end of the sequence resulting in two extra base-pairs. Note that supplementary materials (Table S2) also includes the opening profiles for these two additional base-pairs.

## Electronic supplementary material

Supplementary Tables S1-S5
